# A Child With Abnormal Ocular Ultrasound After Out-of-Hospital Cardiac Arrest

**DOI:** 10.1016/j.acepjo.2026.100419

**Published:** 2026-05-14

**Authors:** Vladimir L. Cousin, Emmanuel Barreau, Ramy Charbel

**Affiliations:** 1 Pediatric Intensive Care, Neonatal Medicine and Emergency, AP-HP Paris Saclay University, Bicêtre Hospital, Le Kremlin-Bicêtre, France; 2 Department of Ophthalmology, AP-HP Paris Saclay University, Bicêtre Hospital, Le Kremlin-Bicêtre, France

**Keywords:** abusive head trauma, intracranial hypertension, pediatrics

## 1 Case Presentation

**Figure 1:**
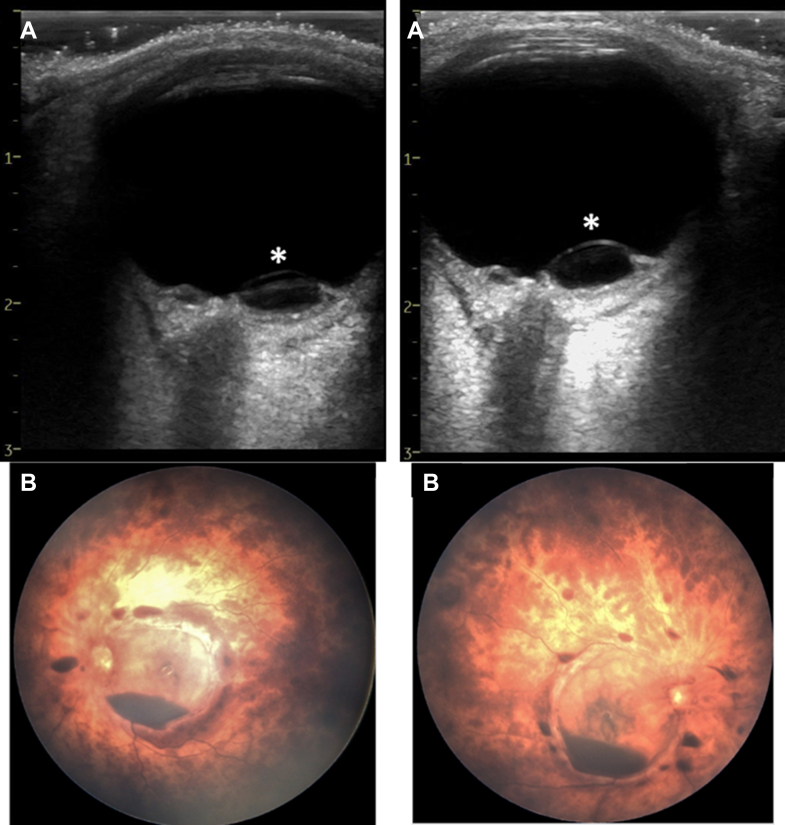
(A) The eye POCUS image with the bulging of the retina posterior wall due to a detachment of the posterior hyaloid called retinoschisis (∗). (B) The corresponding fundoscopic exam confirming the retrohyaloidal hemorrhage with a blood level and retinal fold. POCUS, point-of-care ultrasound.

A 5-month-old patient was admitted to the pediatric intensive care unit (PICU) after an out-of-hospital cardiac arrest. Return to spontaneous circulation was achieved after 30 minutes of cardiopulmonary resuscitation and 2 adrenaline doses. Upon PICU admission, a point-of-care ultrasound assessment, including ocular ultrasound for optic nerve sheath measurement, was performed. It showed an abnormal image with a bulge of the retina’s posterior wall, without optic nerve edema (Fig. 1). A fundoscopic examination confirmed the suspicion of retinal lesions with a retrohyaloidal hemorrhage, highly suspicious of abusive head trauma (AHT).

## 2 Diagnosis: Abusive Head Trauma

Early clinical signs of AHT often include the presence of severe intracranial hypertension with potential additional extracranial traumatic lesions. Children admitted to PICU in this context have a severe prognosis with a mortality rate of 50%.^5^^,^^6^

AHT is a challenge to diagnose, with frequent delayed diagnosis.^1^ With a rapid and noninvasive evaluation of the retina and vitreous, ocular point-of-care ultrasound has interesting characteristics in a situation potentially related to AHT. First, it has excellent diagnostic performance with a sensitivity (96%) and a specificity (100%).^2^ Second, it may give the opportunity of an early diagnosis or suspicion of AHT, nearly 2 days before the fundoscopic examination.^2^ The images pointing to AHT are the presence of retinal hemorrhage, defined as a smooth area of hyperechoic retina protruding in the vitreous; vitreous haemorrhages with the presence of hyperechoic material shifting with eye movements; and retinoschisis, a dome of several layers of the retina, bulging in the vitreous.^2^, ^3^, ^4^

## Funding and Support

By *JACEP Open* policy, all authors are required to disclose any and all commercial, financial, and other relationships in any way related to the subject of this article as per ICMJE conflict of interest guidelines (see www.icmje.org). The authors have stated that no such relationships exist.

## Conflict of Interest

All authors have affirmed they have no conflicts of interest to declare.
